# Editorial: New Boundaries Between Aging, Cognition, and Emotions

**DOI:** 10.3389/fpsyg.2018.00973

**Published:** 2018-06-13

**Authors:** Rocco Palumbo, Alberto Di Domenico

**Affiliations:** ^1^Department of Neurology, Boston University, Boston, MA, United States; ^2^Università degli Studi G. d'Annunzio Chieti e Pescara, Chieti, Italy

**Keywords:** aging, emotions, cognition, lifespan, positivity effect

Numerous studies have reported age-related differences for emotional information (Carstensen et al., 2003; Di Domenico et al., 2016; Mammarella et al., 2017). For example, when, compared to younger adults, older adults reveal a relative preference in attention and memory for positive over negative information (Charles and Carstensen, 2010; Fairfield et al., 2015; Palumbo et al., 2015, 2018; Palumbo et al.). One explanation places emphasis on an emotion processing preference in older adults that reflects their socioemotional self-relevant goals (Custers and Aarts, 2005). Based on evidence from behavioral and neuroscientific research, researchers have realized that it is necessary to propose a new conceptual framework to describe the relationship between cognition and emotion.

Given the growing body of research focused on the interaction between emotions and cognition, the purpose of this research topic is to provide a picture of the state of the art of the interaction between aging, cognition and emotions.

The 12 articles composing this unique Frontiers Research Topic bring together experimental and theoretical research, linking state-of-the-art knowledge about the role of emotions on cognition in the lifespan. Gerino et al. investigate the way in which psychological factors—such as loneliness, resilience, and mental states, in terms of depression and anxiety symptoms—affect the perceived Quality of Life among elderly individuals.

Choi et al. provide converging evidence that social relatedness plays a significant role in physical health, particularly in the older population. Mestre et al. explore the relationship between both, emotional regulation abilities and strategies, and resilience in a sample of adolescents from suburbs high-schools. The study also examines how using different emotional regulation strategies may help the development of resilience levels at this stage.

Di Crosta and La Malva discuss the experience of passage of time in young and elderly adults. In a review of Liebherr et al. the authors present summarize neuropsychological and neurophysiological findings of age-related differences in decision making under ambiguous and objective risk. In this context, the relevance of learning, but also of cognitive and emotional contributors—responsible for age-related differences in decision making—are additionally pointed out. Walther et al. suggested that, steroid hormones and age-related alterations in secretion patterns have been shown to play a crucial role in age-related changes in emotional experience. García-Bajos et al. examined the age-based positivity effect of recall for future positive and negative autobiographical events in young and older adults. Kappes et al. present findings that are consistent with the idea that age-related changes in the processing of emotional information support older adults' general emotional well-being. Deng et al. present the development of a new standardized emotional film database for Asian culture using eight kinds of emotion: fear, disgust, anger, sadness, neutrality, surprise, amusement and pleasure.

Palumbo et al. based on previous research that has shown effects of facial appearance on trait impressions and group stereotypes, investigate the contribution of resemblance to emotion expressions and attractiveness to younger adults' (YA) and older adults' (OA) age and gender stereotypes on the dimensions of warmth and competence. Liao et al. use event-related potentials (ERPs) to investigate the effects of age on neural temporal dynamics of processing task-relevant facial expressions and their relationship to cognitive functions. Finally, Hu et al. provided the first evidence of the Thin-slice Wisdom Paradigm's reliability, its immunity to social desirability, and its validity for assessing candidates' wisdom within a short timeframe.

Altogether, the contributions of this research topic, highlight the crucial role that emotions play on cognition highlighting their importance in the lifespan.

## Author contributions

All authors listed have made a substantial, direct and intellectual contribution to the work, and approved it for publication.

### Conflict of interest statement

The authors declare that the research was conducted in the absence of any commercial or financial relationships that could be construed as a potential conflict of interest.
